# Seasonal optimization and analysis of an Off-grid hybrid renewable energy system for a coastal hotel

**DOI:** 10.1038/s41598-026-51538-3

**Published:** 2026-05-13

**Authors:** Hady S. Abdel Hafez, Saber. M. Saleh, Mokhtar Said

**Affiliations:** Electrical Engineering Department, Faculty of Engineering, Fayoum University, Fayoum, 43518 Egypt

**Keywords:** Hybrid system, Sustainable energy, PV–Wind, Optimization, Energy science and technology, Engineering, Environmental sciences

## Abstract

The world is increasingly shifting toward sustainable and renewable energy systems due to limited fossil fuel sources as well as their negative environmental impacts. Remote coastal tourism facilities face additional challenges because of the high fuel transportation and operation costs. This study focuses on a seasonal analysis and optimization of an off-grid hybrid renewable energy system comprising photovoltaic panels, wind turbines, and a battery energy storage system designed to supply a large five-star hotel located in Safaga, Egypt. Four advanced metaheuristic optimization techniques are used to minimize the cost of energy while maintaining system reliability. Unlike conventional annual sizing approaches, the proposed analysis evaluates the system on a seasonal basis using hourly load and weather data, and compares the optimization algorithms under the same framework to examine their effect on system sizing and cost performance. The analysis results indicate that Fungal Growth Optimizer (FGO) provides competitive solution quality with satisfactory computational performance compared to the other algorithms and the system achieves its minimum Levelized Cost of Energy (LCOE) of 0.098854 USD/kWh during the summer season and the maximum value of 0.159970 USD/kWh occurs in autumn. In summary, the findings confirm that the proposed sustainable system yields a reliable and cost-effective solution in addition to reducing carbon emissions in the Red Sea region.

## Introduction

The consumption of fossil fuels has risen due to population growth and technological advancements accompanied by negative environmental effects including global warming and climate change^1–3^. To reduce reliance on fossil fuels, researchers are exploring alternatives such as optimizing conventional power generation^4–6^, developing cleaner energy generation methods similar to fuel cells^7–9^, and expanding the use of renewable energy systems (RES)^10–12^. Of these possibilities, RES are regarded as the most promising since they can substantially decrease or even remove reliance on fossil fuels. Renewable energy technologies have expanded rapidly in recent years and are now frequently employed for large-scale electricity generation including solar-generated energy^13,14^, wind-generated energy^15–17^, biofuel energy^18,19^, and ocean-derived energy^20,21^.

Weather factors affect how much electricity is produced from RES such as wind and solar. For example, on calm days, wind turbines may produce no power at all, but on cloudy days, solar energy generation facilities can lose most of their output^22^. The inherent unpredictability of RES increases the importance of accurate forecasting and operational planning^23^. In addition, their intermittent nature necessitates integration with other renewable sources as well as appropriate energy storage technologies^24,25^. Hydrogen-based energy storage has also been explored as an effective solution in hybrid multi-energy systems, particularly through the integration of electrolyzer and fuel cell technologies^26^. Flexibility in the power system is defined as the ability of the system to balance generated power with demand loads while ensuring a continuous flow of electricity, even in unpredictable conditions^27^.

With rising electricity demand and growing environmental concerns, RES are becoming increasingly important for their sustainability and potential to reduce dependence on fossil fuels. Early studies focused on using either wind or solar alone^28,29^, then shifted to hybrid systems combining both for more reliability and efficiency because each source alone faces challenges due to its unpredictable conditions, such as no sunlight at night and changing wind speeds, which impact power stability. To address this, Hybrid Renewable Energy Systems (HRES) integrate various resources like solar, wind, and sometimes diesel generators or grid connections to ensure more reliable and cost-effective electricity^30–34^. Recent research efforts have been directed toward enhancing the performance of HRES and reducing energy generation costs through advanced technologies and optimization methods^35^. Table 1 presents a comparison of these studies, detailing their years, locations, case studies, system types, components, tools, and results^36–46^.

**Table 1:** Comparison of recent related studies.

Ref	Year	Location	Case Study	Type	Systems	Tools	Results
^46^	2026	Gaza City	Hybrid microgrid system	Off-grid	PV-WT-BES	metaheuristic algorithms	LCOE of 0.0923 USD/kWh
^45^	2025	Hurghada, Egypt	Hospital	On-grid	PV-WT-BES	metaheuristic algorithms	LCOE of 0.0347 USD/kWh NPC of 3.75 M USD
^44^	2025	New Administrative Capital, New Cairo, Egypt	An international school	Off-grid	PV-WT-DG- BES -FC-ELZ-HT	(HOMER) software	LCOE of 0.153 USD/kWh TNPC of 1.77 M USD
^43^	2025	Kakdwip subdivision, India	A remote village (Gobardhanpur)	Off-grid	PV-WT-BG-BES	Machine learning techniques	LCOE of 0.278 USD/kWh NPC of 1.61 M USD
^42^	2025	Dakhla, Morocco	Different locations in Morocco	On-grid	PV and wind	metaheuristic algorithms	LCOE of 0.0662 USD/kWh
^40^	2025	Ghana	Industrial electrification	On-grid	SMR-PV-WT-BES	HOMER Pro software	LCOE of 0.185 USD/kWh
^39^	2024	Thala City, Tunisia	Remote rural electrification in the highest region in Tunisia.	off-grid and on-grid	Wind/BG/PHS/BES	(HOMER) software	LCOE of 0.042 USD/kWh NPC of 501,540 USD
^41^	2024	Qena and Red Sea, Egypt	Rural electrification in a village	Off-grid	PV-WT-DG- BES	HOMER Pro software	Qena LCOE of 0.212 USD/kWh NPC of 665,267 USD Red Sea LCOE of 0.181 USD/kWh NPC of 569,353 USD
^38^	2022	Djelfa, Algeria	Fifteen residential housing units	Off-grid	PV-WT-DG- BES	metaheuristic algorithms	LCOE of 0.255 USD/kWh
^37^	2022	Zambia	Rural electrification in sub-Saharan Africa: A case study of Chilubi Island, a remote district without electricity.	Off-grid	PV-DG-BES	(HOMER) software	LCOE of 0.182 USD/kWh
^36^	2020	southern Ecuador	National University of Education		PV-WT-HKT -BES-DG	HOMER Pro software	LCOE of 0.88 USD/kWh

Although the studies summarized in Table 1 provide valuable insights into the design and optimization of HRES, most of them focus on general electrification scenarios or annual techno-economic assessment. In addition, limited attention has been given to large hospitality facilities with highly variable seasonal demand, particularly in coastal areas where both renewable resource availability and electrical load profiles change significantly over the year^47,48^.

Despite the progress achieved in renewable energy technologies, challenges still remain in developing optimization frameworks that can adequately capture the variability of renewable resources, seasonal changes in load demand, and system operational constraints. This issue becomes more critical in coastal hospitality applications, where electricity demand is not constant throughout the year and is strongly affected by seasonal occupancy and weather conditions. Accordingly, this study develops a techno-economic optimization framework for an off-grid hybrid photovoltaic (PV)–wind turbine (WT)–battery energy storage (BES) system designed to supply a five-star hotel located in Safaga by using advanced optimization techniques to minimize the Levelized Cost of Energy (LCOE) while ensuring system reliability. The proposed framework is based on the hotel’s 8760-hour load profile and local meteorological data, while explicitly evaluating the system on a seasonal basis rather than relying only on an annual aggregated assessment. In addition, four metaheuristic optimization algorithms, namely Genetic Algorithm (GA), Grey Wolf Optimizer (GWO), Cuckoo Search (CS), and Fungal Growth Optimizer (FGO), are implemented within the same MATLAB environment and under the same technical and economic assumptions. This allows not only the identification of the optimal system size for each season, but also a consistent comparison of the algorithms in terms of solution quality and computational performance.

## Modeling of the hybrid system components

**Fig. 1:**
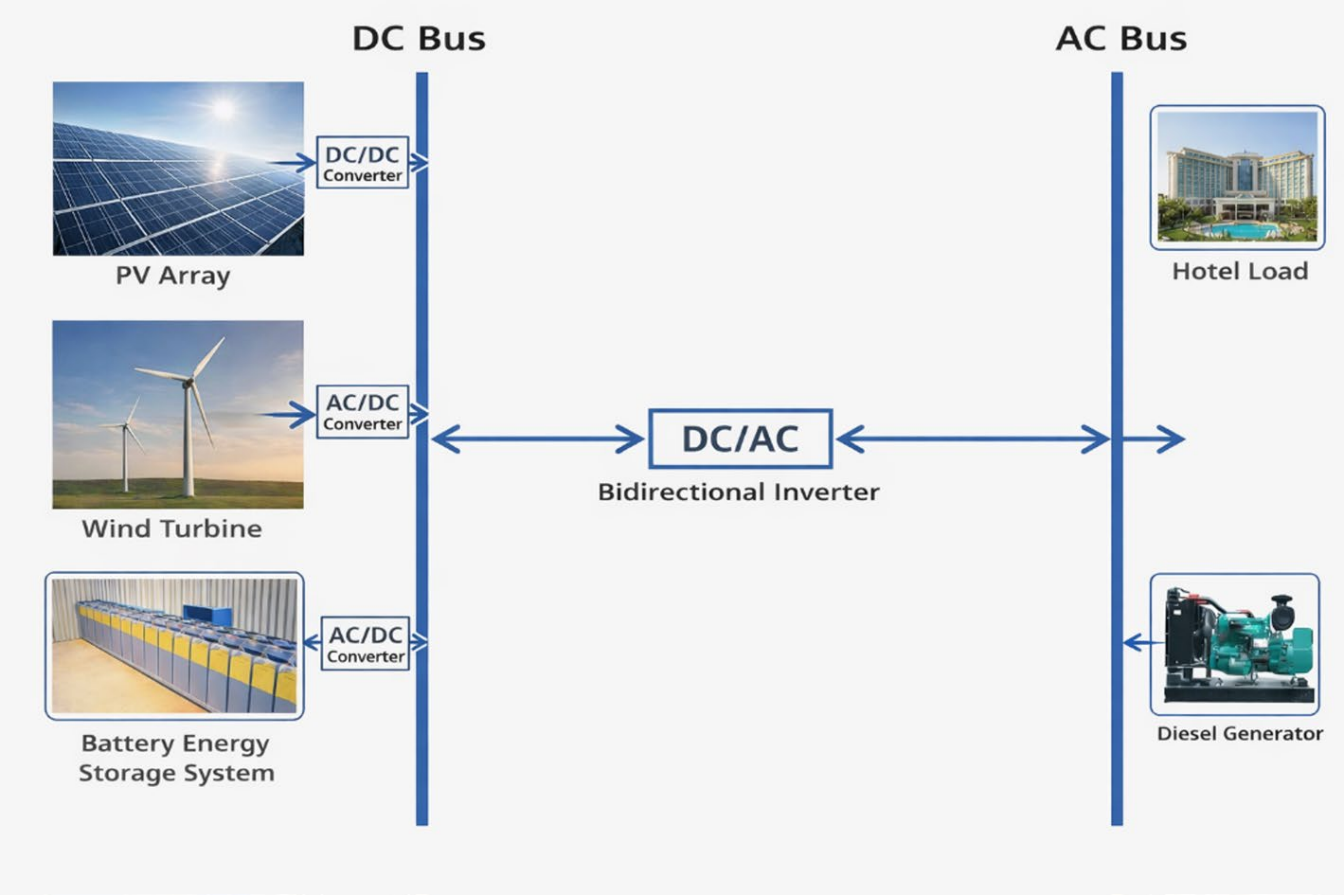
Schematic architecture of the proposed hybrid renewable energy system.

HRES comprises PV arrays generating DC power, WTs producing AC power, a BES storing DC power and a diesel generator (DG) acting as a backup source for emergency and life safety loads in worst case conditions, all integrated through DC/AC buses by inverters and converters, as shown in Fig. 1. All RES units are connected to the DC bus through converters. The DC/AC buses are interconnected through an inverter to convert the generated power into the AC power required by the load.

### Photovoltaic energy system design

The PV modules are created by connecting PV cells to generate the required output voltage, current and power for each module. Many models were created to compute the output power of a PV panel. This research presents a straightforward model that depends only on two factors: solar irradiation and ambient temperature.

The output power of this model can be determined using Eq. (1)^49^

1 $$\:{\mathrm{P}}_{\mathrm{p}\mathrm{v}\_\mathrm{o}\mathrm{u}\mathrm{t}}\left(\mathrm{t}\right)={\mathrm{P}}_{\mathrm{p}\mathrm{v}\_\mathrm{r}}\times\:{\mathrm{f}}_{\mathrm{p}\mathrm{v}}\times\:\frac{{\mathrm{G}}_{\mathrm{t}}\left(\mathrm{t}\right)}{{\mathrm{G}}_{\mathrm{t}\_\mathrm{S}\mathrm{T}\mathrm{C}}}\times\:\left[1+{{\upalpha\:}}_{\mathrm{t}}\left({\mathrm{T}}_{\mathrm{C}}\left(\mathrm{t}\right)-\:{\mathrm{T}}_{{\mathrm{C}}_{\mathrm{S}\mathrm{T}\mathrm{C}}}\right)\right]$$

where: $\:{\mathrm{P}}_{\mathrm{p}\mathrm{v}\_\mathrm{o}\mathrm{u}\mathrm{t}}\left(\mathrm{t}\right)$ refers to the generated power while $\:{\mathrm{P}}_{\mathrm{p}\mathrm{v}\_\mathrm{r}}$ refers to the rated power (W) at $\:\mathrm{S}\mathrm{T}\mathrm{C}$, $\:{\mathrm{f}}_{\mathrm{p}\mathrm{v}}$ is the PV derating factor [%], $\:{\mathrm{G}}_{\mathrm{t}}\left(\mathrm{t}\right)$ refers to the solar flux while $\:{\mathrm{G}}_{\mathrm{t}\_\mathrm{S}\mathrm{T}\mathrm{C}}$ refers to the solar flux at STC $\:({\mathrm{G}}_{\mathrm{t}\_\mathrm{S}\mathrm{T}\mathrm{C}}=1000\:\mathrm{W}/{m}^{2})$, $\:{{\upalpha\:}}_{\mathrm{t}}$ represents the coefficient of temperature and its value is $\:{{\upalpha\:}}_{\mathrm{t}}\:=\:-3.7\:\times\:{10}^{-3}/\mathrm{C}^\circ\:$ for (Si) solar cells, $\:{\mathrm{T}}_{\mathrm{C}}\left(\mathrm{t}\right)$ refers to the cell ambient temperature while $\:{\mathrm{T}}_{\mathrm{C}\_\mathrm{S}\mathrm{T}\mathrm{C}}$ is the cell temperature at STC $\:({\mathrm{T}}_{\mathrm{C}\_\mathrm{S}\mathrm{T}\mathrm{C}}=25\:^\circ\mathrm{C})$. The cell temperature is determined by Eq. (2)^50^

2 $$\:{\mathrm{T}}_{\mathrm{C}}\:=\:{\mathrm{T}}_{\mathrm{a}}\:+\left[\frac{\mathrm{N}\mathrm{O}\mathrm{C}\mathrm{T}-20}{800}\right]\:\mathrm{*}{\mathrm{G}}_{\mathrm{T}}\:\:$$

where: $\:{\mathrm{T}}_{\mathrm{a}}$ refers to the ambient temperature in $\:(\mathrm{C}^\circ\:)$ and NOCT refers to the nominal operating temperature of cell ($\:45\:\mathrm{C}^\circ\:$ to $\:47\:\mathrm{C}^\circ\:$).

### Wind energy system design

The power produced by WT changes depending on how fast the wind is blowing. Wind speed changes with height, so the measured wind speed needs to match the turbine’s hub height. Two models are commonly used to describe this: the log-law and the power law. This study uses the power law model, which is more suitable for predicting wind speed in the region, as shown in Eq. (3)^51^

3 $$\:\frac{{\mathrm{V}}_{2}}{{\mathrm{V}}_{1}}={\left(\frac{{\mathrm{h}}_{2}}{{\mathrm{h}}_{1}}\right)}^{{\upalpha\:}}\:$$

Where: $\:{\mathrm{V}}_{2}(\mathrm{m}/\mathrm{s})$ refers to the speed of wind at the hub height$\:\:{\mathrm{h}}_{2}\left(\mathrm{m}\right)$ while $\:{\mathrm{V}}_{1}(\mathrm{m}/\mathrm{s})$ refers to the speed of wind at the reference height$\:\:\mathrm{h}1\left(\mathrm{m}\right)$, $\:{\upalpha\:}$ represents the friction coefficient that depends on several variable factors such as wind speed, terrain roughness, hub height, temperature, time of day, and season. Its value of ($\:{\upalpha\:}=\frac{1}{7}$) is generally accepted. The generated power of the WT can be determined using Eq. (4)^51^

4 $$\:{\mathrm{P}}_{\mathrm{w}\mathrm{t}}\left(\mathrm{t}\right)=\:\:\left\{\begin{array}{c}\:\:\:\:\:\:\:\:\:\:{\:\mathrm{P}}_{\mathrm{r}}\:\:\:\:\:\:\:\:\:\:\:\:\:\:\:\:\:\:\:\:\:\:\:\:\:\:\:\:\:\:\:\:\:\:\:\:\:\:\:\:\:\:\:\:\:\:\:\:\:\:\:\:\:\:\:\:\:\:\:\:\:\:\:\:\:\:\:\:\:{\mathrm{v}}_{\mathrm{r}}\le\:\:v\:\le\:\:{\mathrm{v}}_{\mathrm{c}\mathrm{u}\mathrm{t}\:\mathrm{o}\mathrm{f}\mathrm{f}}\\\:{\mathrm{v}}^{3}\left(\frac{{\mathrm{P}}_{\mathrm{r}}}{{\mathrm{v}}_{\mathrm{r}}^{3}-{\mathrm{v}}_{\mathrm{c}\mathrm{u}\mathrm{t}\_\mathrm{i}\mathrm{n}}^{3}}\right)-{\mathrm{P}}_{\mathrm{r}}\left(\frac{{\mathrm{v}}_{\mathrm{c}\mathrm{u}{\mathrm{t}}_{\mathrm{i}\mathrm{n}}}^{3}}{{\mathrm{v}}_{\mathrm{r}}^{3}-{\mathrm{v}}_{\mathrm{c}\mathrm{u}{\mathrm{t}}_{\mathrm{i}\mathrm{n}}}^{3}}\right)\:\:\:\:\:\:\:\:\:\:\:\:\:\:{\mathrm{v}}_{\mathrm{c}\mathrm{u}\mathrm{t}\:\mathrm{i}\mathrm{n}}\:\le\:\:v\:\le\:\:{\mathrm{v}}_{\mathrm{r}}\:\\\:\:\:\:0\:\:\:\:\:\:\:\:\:\:\:\:\:\:\:\:\:\:\:\:\:\:\:\:\:\:\:\:\:\:\:\:\:\:\:\:\:\:\:\:\:\:\:\:\:\:\:\:\:\:\:\:\:\:\:\:\:\:\:\:\:\:\:\:\:\:\:\:\:\:\:\:\:\:\:\:\:otherwise\end{array}\right.\:\:\:$$

**Fig. 2:**
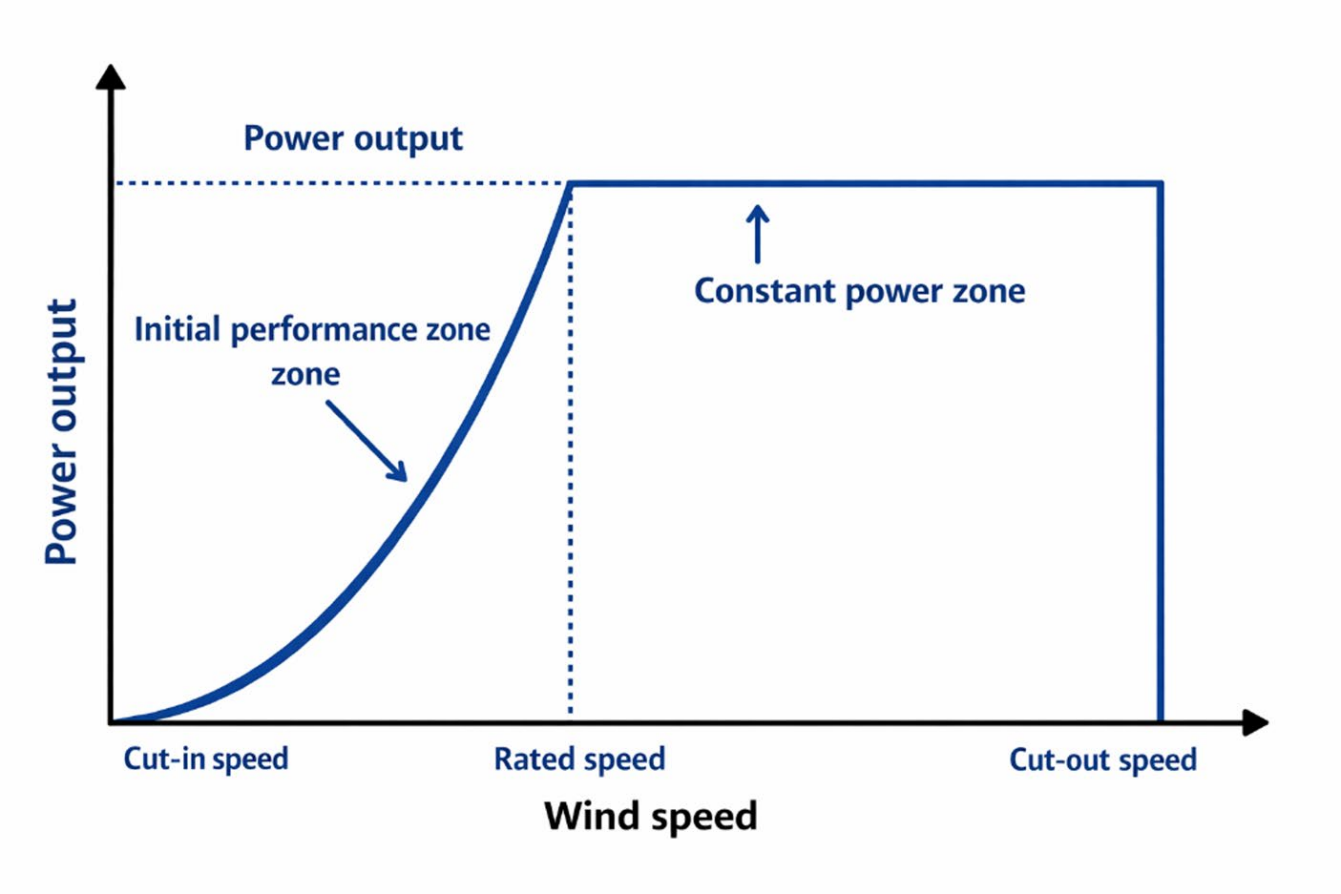
Performance characteristic curve of a wind turbine.

Where: $\:{\mathrm{P}}_{\mathrm{r}}$ refers to the rated power $\:\left(\mathrm{k}\mathrm{W}\right)$, $\:\mathrm{v}$ represents the actual speed of wind while $\:{\mathrm{v}}_{\mathrm{c}\mathrm{u}\mathrm{t}\_\mathrm{i}\mathrm{n}}$,$\:\:{\mathrm{v}}_{\mathrm{r}}$, $\:{\mathrm{v}}_{\mathrm{c}\mathrm{u}\mathrm{t}\_\mathrm{o}\mathrm{u}\mathrm{t}}$ represent the cut-in, rated and cut-out specific wind speed of the WT in (m/s) respectively. Figure 2 shows the output power of the WT versus the speed of wind^51^. 

The $\:{\mathrm{P}}_{\mathrm{r}}$ of a WT can be determined using Eq. (5)^38^

5 $$\:{\mathrm{P}}_{\mathrm{r}}=\frac{1}{2}{\mathrm{C}}_{\mathrm{p}}\times\:{{\uprho\:}}_{\mathrm{a}\mathrm{i}\mathrm{r}}\times\:{\mathrm{A}}_{\mathrm{w}\mathrm{i}\mathrm{n}\mathrm{d}}\times\:{\mathrm{v}}_{\mathrm{r}}^{3}\:$$

Where: $\:{\mathrm{A}}_{\mathrm{w}\mathrm{i}\mathrm{n}\mathrm{d}}$ refers to the area swept by the blades, $\:{\mathrm{C}}_{\mathrm{p}}$ refers to the maximum power coefficient and $\:{{\uprho\:}}_{\mathrm{a}\mathrm{i}\mathrm{r}}$ refers to the air density.

### Battery energy storage system design

In this system, the HRES utilizes a BES to store excess energy for use during periods when HRES generation isn’t sufficient for the required load or RES are unavailable. The capacity of the BES can be determined using Eq. (6)^52^

6 $$\:{\mathrm{B}\mathrm{T}}_{\mathrm{c}}=\frac{\mathrm{A}\mathrm{D}\times\:{\mathrm{E}}_{\mathrm{L}}}{{{\upeta\:}}_{\mathrm{I}\mathrm{N}\mathrm{V}}\times\:{{\upeta\:}}_{\mathrm{B}\mathrm{T}}\times\:\mathrm{D}\mathrm{O}\mathrm{D}}$$

Where: $\:{\mathrm{E}}_{\mathrm{L}}$ refers to the demand load, $\:\mathrm{D}\mathrm{O}\mathrm{D}$ refers to the BES depth of discharge, $\:{{\upeta\:}}_{\mathrm{I}\mathrm{N}\mathrm{V}}\:$and $\:{{\upeta\:}}_{\mathrm{B}\mathrm{T}}\:$ are the efficiencies and the $\:\mathrm{A}\mathrm{D}$ represents the critical autonomy days.

The state of energy (SOE) of the BES, regarding its discharging and charging states, should be maintained within the specified limits of $\:{\mathrm{S}\mathrm{O}\mathrm{E}}_{\mathrm{m}\mathrm{a}\mathrm{x}}$ and $\:{\mathrm{S}\mathrm{O}\mathrm{E}}_{\mathrm{m}\mathrm{i}\mathrm{n}}$ as illustrated in (7) and (8) to prolong the operational lifespan of the BES.

7 $$\:{\mathrm{S}\mathrm{O}\mathrm{E}}_{\mathrm{m}\mathrm{i}\mathrm{n}}\:\le\:\:\mathrm{S}\mathrm{O}\mathrm{E}\left(\mathrm{t}\right)\:\le\:\:{\mathrm{S}\mathrm{O}\mathrm{E}}_{\mathrm{m}\mathrm{a}\mathrm{x}}\:$$

8 $$\:(1\:-\:\mathrm{D}\mathrm{O}\mathrm{D})\:{\mathrm{B}\mathrm{T}}_{\mathrm{c}}\:\le\:\:\mathrm{S}\mathrm{O}\mathrm{E}\left(\mathrm{t}\right)\:\le\:\:{\mathrm{B}\mathrm{T}}_{\mathrm{c}}\:\:$$

The maximum energy state of the battery is equal to the sum of the DOD energy and the minimum energy state.

### Diesel generator design

The DG is therefore not intended to operate as a primary generation source during normal conditions. Its role is limited to backup support during rare periods of insufficient renewable generation and low battery state, particularly to maintain critical and life-safety loads. Accordingly, the system philosophy remains based on maximizing renewable energy utilization while retaining a practical backup option to enhance supply security under worst case scenarios.

The hourly fuel usage of DG can be determined using Eq. (9)^52^

9 $$\:{\mathrm{F}}_{\mathrm{D}\mathrm{G}}\left(\mathrm{t}\right)={\upalpha\:}{\mathrm{P}}_{\mathrm{D}\mathrm{G}}\left(\mathrm{t}\right)+{\upbeta\:}{\mathrm{P}}_{\mathrm{r}\:\:\:\:\:\:\:\:}$$

Where: $\:{\mathrm{F}}_{\mathrm{D}\mathrm{G}}\left(\mathrm{t}\right)$ refers to the fuel consumption ($\:\mathrm{L}/\mathrm{h}\mathrm{r}$), $\:{\mathrm{P}}_{\mathrm{D}\mathrm{G}}\left(\mathrm{t}\right)$ refers to the generated power while $\:{\mathrm{P}}_{\mathrm{r}}$ refers to the generator capacity (kW). $\:{\upalpha\:}$ represents the fuel curve slope coefficient ($\:\mathrm{L}/\mathrm{h}\mathrm{r}/\mathrm{k}\mathrm{W}\mathrm{o}\mathrm{u}\mathrm{t}\mathrm{p}\mathrm{u}\mathrm{t}$) while $\:{\upbeta\:}$ represents the fuel intercept coefficient ($\:\mathrm{L}/\mathrm{h}\mathrm{r}/\mathrm{k}\mathrm{W}\mathrm{r}\mathrm{a}\mathrm{t}\mathrm{e}\mathrm{d}$). The values of $\:{\upalpha\:}$ and $\:{\upbeta\:}$ used in this study are $\:{\upalpha\:}=0.246\:\:$and $\:{\upbeta\:}=\:0.08415\:$.

The efficiency of the DG can be determined using Eq. (10)^53^

10 $$\:{{\upeta\:}}_{\mathrm{o}\mathrm{v}\mathrm{e}\mathrm{r}\mathrm{a}\mathrm{l}\mathrm{l}}\:=\:{{\upeta\:}}_{\mathrm{b}\mathrm{r}\mathrm{a}\mathrm{k}\mathrm{e}\_\mathrm{t}\mathrm{h}\mathrm{e}\mathrm{r}\mathrm{m}\mathrm{a}\mathrm{l}}\times\:\:{{\upeta\:}}_{\mathrm{g}\mathrm{e}\mathrm{n}\mathrm{e}\mathrm{r}\mathrm{a}\mathrm{t}\mathrm{o}\mathrm{r}}\:\:\:\:\:\:\:\:$$

where: $\:{{\upeta\:}}_{\mathrm{o}\mathrm{v}\mathrm{e}\mathrm{r}\mathrm{a}\mathrm{l}\mathrm{l}}$, $\:{{\upeta\:}}_{\mathrm{g}\mathrm{e}\mathrm{n}\mathrm{e}\mathrm{r}\mathrm{a}\mathrm{t}\mathrm{o}\mathrm{r}}$ and $\:{{\upeta\:}}_{\mathrm{b}\mathrm{r}\mathrm{a}\mathrm{k}\mathrm{e}\_\mathrm{t}\mathrm{h}\mathrm{e}\mathrm{r}\mathrm{m}\mathrm{a}\mathrm{l}}$ refer to the overall, generator and brake thermal efficiencies, respectively.

### Inverter and converter design

These are power devices that convert electrical power between AC and DC, enabling effective energy transfer among RES, BES and the load. The bidirectional power flow between the AC and DC buses is provided through the inverter/converter, as shown in Fig. 1. The inverter/converter model is developed based on the expected load variations, power surges, and system performance. The inverter capacity is determined by the peak load demand ($\:{\mathrm{P}}_{\mathrm{L}}^{\mathrm{P}\mathrm{e}\mathrm{a}\mathrm{k}}$).

The rated power of the inverter can be determined using Eq. (11)^54^:

11 $$\:{\mathrm{P}}_{\mathrm{I}\mathrm{N}\mathrm{V}}\:=\:\frac{{\mathrm{P}}_{\mathrm{L}}^{\mathrm{P}\mathrm{e}\mathrm{a}\mathrm{k}}}{{{\upeta\:}}_{\mathrm{I}\mathrm{N}\mathrm{V}}}\:$$

## Economic analysis of the hybrid system

The cost model for each HRES component includes the initial capital cost ($\:\mathrm{I}\mathrm{C}$), the replacement cost ($\:\mathrm{R}\mathrm{C})$ and operating and maintenance costs ($\:\mathrm{O}\&\mathrm{M}\mathrm{C}$) which contribute to the total net present cost ($\:\mathrm{T}\mathrm{C}$), calculated during the system’s full lifecycle. The $\:\mathrm{T}\mathrm{C}$ for each component can be determined using the following Eqs. (12 − 21)^46^

12 $$\:{\mathrm{T}\mathrm{C}}_{\mathrm{P}\mathrm{V}}\:={\mathrm{I}\mathrm{C}}_{\mathrm{P}\mathrm{V}}\:+{\mathrm{O}\&\mathrm{M}\mathrm{C}}_{\mathrm{P}\mathrm{V}}\:=\:{\mathrm{N}}_{\mathrm{P}\mathrm{V}}\:\times\:\left[{\mathrm{I}\mathrm{N}\mathrm{P}}_{\mathrm{P}\mathrm{V}}\:+{\sum\:}_{\mathrm{n}=1}^{\mathrm{n}=25}\frac{{\mathrm{O}\&\mathrm{M}\mathrm{P}}_{\mathrm{P}\mathrm{V}}}{{(1+\mathrm{r})}^{\mathrm{n}-1}}\right]\:$$

13 $$\:{\mathrm{T}\mathrm{C}}_{\mathrm{W}\mathrm{T}}\:={\mathrm{I}\mathrm{C}}_{\mathrm{W}\mathrm{T}}\:+{\mathrm{O}\&\mathrm{M}\mathrm{C}}_{\mathrm{W}\mathrm{T}}\:=\:{\mathrm{N}}_{\mathrm{W}\mathrm{T}}\:\times\:\left[{\mathrm{I}\mathrm{N}\mathrm{P}}_{\mathrm{W}\mathrm{T}}\:+{\sum\:}_{\mathrm{n}=1}^{\mathrm{n}=25}\frac{{\mathrm{O}\&\mathrm{M}\mathrm{P}}_{\mathrm{W}\mathrm{T}}}{{(1+\mathrm{r})}^{\mathrm{n}-1}}\right]\:$$

14 $$\:{\mathrm{T}\mathrm{C}}_{\mathrm{B}\mathrm{T}}\:={\mathrm{I}\mathrm{C}}_{\mathrm{B}\mathrm{T}}\:+{\mathrm{R}\mathrm{C}}_{\mathrm{B}\mathrm{T}}+{\mathrm{O}\&\mathrm{M}\mathrm{C}}_{\mathrm{B}\mathrm{T}}\:\:$$

15 $$\:{\mathrm{T}\mathrm{C}}_{\mathrm{B}\mathrm{T}}\:=\:{\mathrm{N}}_{\mathrm{B}\mathrm{T}}\:\times\:\left[{\mathrm{I}\mathrm{N}\mathrm{P}}_{\mathrm{B}\mathrm{T}}\:+\frac{{\mathrm{R}\mathrm{P}}_{\mathrm{B}\mathrm{T}}}{{(1+\mathrm{r})}^{{\mathrm{L}\mathrm{S}}_{\mathrm{B}\mathrm{T}}}}+{\sum\:}_{\mathrm{n}=1}^{\mathrm{n}=25}\frac{{\mathrm{O}\&\mathrm{M}\mathrm{P}}_{\mathrm{B}\mathrm{T}}}{{(1+\mathrm{r})}^{\mathrm{n}-1}}\right]\:$$

16 $$\:{\mathrm{T}\mathrm{C}}_{\mathrm{I}\mathrm{N}\mathrm{V}}\:={\mathrm{I}\mathrm{C}}_{\mathrm{I}\mathrm{N}\mathrm{V}}\:+{\mathrm{R}\mathrm{C}}_{\mathrm{I}\mathrm{N}\mathrm{V}}+{\mathrm{O}\&\mathrm{M}\mathrm{C}}_{\mathrm{I}\mathrm{N}\mathrm{V}}\:\:$$

17 $$\:{\mathrm{T}\mathrm{C}}_{\mathrm{I}\mathrm{N}\mathrm{V}}\:=\:{\mathrm{N}}_{\mathrm{I}\mathrm{N}\mathrm{V}}\:\times\:\left[{\mathrm{I}\mathrm{N}\mathrm{P}}_{\mathrm{I}\mathrm{N}\mathrm{V}}\:+\frac{{\mathrm{R}\mathrm{P}}_{\mathrm{I}\mathrm{N}\mathrm{V}}}{{(1+\mathrm{r})}^{{\mathrm{L}\mathrm{S}}_{\mathrm{I}\mathrm{N}\mathrm{V}}}}+{\sum\:}_{\mathrm{n}=1}^{\mathrm{n}=25}\frac{{\mathrm{O}\&\mathrm{M}\mathrm{P}}_{\mathrm{I}\mathrm{N}\mathrm{V}}}{{(1+\mathrm{r})}^{\mathrm{n}-1}}\right]$$

18 $$\:{\mathrm{T}\mathrm{C}}_{\mathrm{C}\mathrm{C}}\:={\mathrm{I}\mathrm{C}}_{\mathrm{C}\mathrm{C}}\:+{\mathrm{R}\mathrm{C}}_{\mathrm{C}\mathrm{C}}+{\mathrm{O}\&\mathrm{M}\mathrm{C}}_{\mathrm{C}\mathrm{C}}\:\:\:$$

19 $$\:{\mathrm{T}\mathrm{C}}_{\mathrm{C}\mathrm{C}}\:=\:{\mathrm{N}}_{\mathrm{C}\mathrm{C}}\:\times\:\left[{\mathrm{I}\mathrm{N}\mathrm{P}}_{\mathrm{C}\mathrm{C}}\:+\frac{{\mathrm{R}\mathrm{P}}_{\mathrm{C}\mathrm{C}}}{{(1+\mathrm{r})}^{{\mathrm{L}\mathrm{S}}_{\mathrm{C}\mathrm{C}}}}+{\sum\:}_{\mathrm{n}=1}^{\mathrm{n}=25}\frac{{\mathrm{O}\&\mathrm{M}\mathrm{P}}_{\mathrm{C}\mathrm{C}}}{{(1+\mathrm{r})}^{\mathrm{n}-1}}\right]\:$$

20 $$\:{\mathrm{T}\mathrm{C}}_{\mathrm{D}\mathrm{G}}\:={\mathrm{I}\mathrm{C}}_{\mathrm{D}\mathrm{G}}\:+{\mathrm{O}\&\mathrm{M}\mathrm{C}}_{\mathrm{D}\mathrm{G}}\:+\:\:{\mathrm{F}\mathrm{C}}_{\mathrm{D}\mathrm{G}}\:\:\:\:\:\:$$

21 $$\:{\mathrm{T}\mathrm{C}}_{\mathrm{D}\mathrm{G}}\:=\:{\mathrm{N}}_{\mathrm{D}\mathrm{G}}\:\times\:\left[{\mathrm{I}\mathrm{N}\mathrm{P}}_{\mathrm{D}\mathrm{G}}\:+{\sum\:}_{\mathrm{n}=1}^{\mathrm{n}=25}\frac{{\mathrm{O}\&\mathrm{M}\mathrm{P}}_{\mathrm{D}\mathrm{G}}}{{(1+\mathrm{r})}^{\mathrm{n}-1}}+{\sum\:}_{\mathrm{n}=1}^{\mathrm{n}=25}\frac{{\mathrm{F}\mathrm{C}}_{\mathrm{D}\mathrm{G}}}{{(1+\mathrm{r})}^{\mathrm{n}-1}}\right]\:$$

where: $\:\mathrm{I}\mathrm{N}\mathrm{P}$ refers to the initial capital cost of one unit while $\:\mathrm{N}$ refers to the number of units and $\:\mathrm{n}$ represents the project lifetime. PV, WT, BT, INV, CC and DG denote photovoltaic panel, wind turbine, battery, inverter, charge controller and diesel generator, respectively.

The financial and operational characteristics of all energy resources and system components included in the HRES are summarized in Tables 2, 3, 4, 5 and 6. These parameters are fundamental for determining the system’s operational performance and economic feasibility throughout the projected 25-year lifetime of HRES at the selected site.

**Table 2:** PV array technical specifications and economic assumptions.

Solar Panel (640 W Hyundai Mono TOPCon Bifacial XXL Solar Panel)^55^	
Lifespan	25 Years
Rated capacity (W)	640
PV panel Efficiency (%)	22.9%
Initial cost	300 USD
O&M cost	10 USD

**Table 3:** Wind turbine technical specifications and economic assumptions.

Wind turbine (Aeolos Aeolos-H 50 kW)^56^	
Lifespan	25 Years
Max. hub height	50 m
Rated power	50 kW
Initial cost	130,000 USD
O&M cost	3000 USD

**Table 4:** Inverter technical specifications and economic assumptions.

High-Efficiency 1 MW Hybrid Inverter for off-Grid Solar^57^	
Lifespan	15 Years
Rated power	1000 kW
Initial cost	100,000 USD
O&M cost	2000 USD

**Table 5:** Battery technical specifications and economic assumptions.

Lithium-ion (LiFePO4) Battery^58^	
Declared capacity (kWh)	10
Lifespan	10
Initial cost	2000 USD
O&M cost	20 USD

**Table 6:** Charge controller technical specifications and economic assumptions.

Solar-charge-controller^59^	
Rated power (kW)	5
Lifespan	10
Initial cost	300 USD
O&M cost	10 USD

## Optimization method and strategy

The hybrid system, consisting of PV, WT, BT and a DG, is optimally sized using a combined techno-economic framework. The main goal of the optimization process is to ensure a reliable supply of electrical energy while achieving the lowest possible cost. In this context, the LCOE serves as the main economic performance indicator, whereas the Loss of Power Supply Probability (LPSP) is employed as the principal reliability metric.

### Objective function

The optimization aims to minimize the LCOE while maintaining system reliability. Equation (22) is used to calculate discount rate $\:\mathrm{r}$, which is then used to calculate the capital recovery factor ($\:\mathrm{C}\mathrm{R}\mathrm{F}$).

22 $$\:\mathrm{r}=\frac{\mathrm{i}-\mathrm{f}}{\mathrm{i}+\mathrm{f}}\:$$

Where: $\:\mathrm{f}$ refers to the inflation rate while $\:\mathrm{i}$ refers to the interest rate. Equations (23)–(26) are used to determine the $\:\mathrm{L}\mathrm{C}\mathrm{O}\mathrm{E}$^46^.

23 $$\:\mathrm{C}\mathrm{R}\mathrm{F}=\frac{\mathrm{r}\times\:{(1+\mathrm{r})}^{\mathrm{n}}}{{(1+\mathrm{r})}^{\mathrm{n}}-1}\:$$

24 $$\:\mathrm{T}\mathrm{N}\mathrm{P}\mathrm{C}\:={\mathrm{T}\mathrm{C}}_{\mathrm{P}\mathrm{V}}+{\mathrm{T}\mathrm{C}}_{\mathrm{W}\mathrm{T}}+{\mathrm{T}\mathrm{C}}_{\mathrm{B}\mathrm{T}}\:+\:{\mathrm{T}\mathrm{C}}_{\mathrm{I}\mathrm{N}\mathrm{V}}+\:\:{\mathrm{T}\mathrm{C}}_{\mathrm{C}\mathrm{C}}+\:\:\:{\mathrm{T}\mathrm{C}}_{\mathrm{D}\mathrm{G}}\:\:\:\:\:$$

25 $$\:\mathrm{A}\mathrm{T}\mathrm{C}=\mathrm{C}\mathrm{R}\mathrm{F}\:\:\:\times\:\:\:\:\:\mathrm{T}\mathrm{N}\mathrm{P}\mathrm{C}\:\:\:\:$$

26 $$\:\mathrm{L}\mathrm{C}\mathrm{O}\mathrm{E}=\frac{\mathrm{A}\mathrm{T}\mathrm{C}}{{\mathrm{E}}_{\mathrm{l}\mathrm{o}\mathrm{a}\mathrm{d}\:\mathrm{s}\mathrm{e}\mathrm{r}\mathrm{v}\mathrm{e}\mathrm{d}}}=\frac{\mathrm{A}\mathrm{T}\mathrm{C}}{{\int\:}_{\mathrm{t}=1}^{24}{\mathrm{P}}_{\mathrm{l}\mathrm{o}\mathrm{a}\mathrm{d}}\left(\mathrm{t}\right)\mathrm{d}\mathrm{t}\times\:365}\:$$

where $\:\mathrm{A}\mathrm{T}\mathrm{C}$ is an equivalent annualized total cost which equitably distributes the system’s costs over its entire lifetime, $\:\mathrm{T}\mathrm{N}\mathrm{P}\mathrm{C}$ is the total net present cost and $\:{\mathrm{P}}_{\mathrm{l}\mathrm{o}\mathrm{a}\mathrm{d}}$ represents the hourly load power consumption.

### Constraints

The optimization process of the whole system must be controlled by several system constraints and limitations including operational performance and physical feasibility to ensure the system’s stability. The following constraints are considered in this study. The LPSP indicates the system reliability. The following formulas are used to calculate the LPSP^60^:

27 $$\:\mathrm{L}\mathrm{P}\mathrm{S}\mathrm{P}={\sum\:}_{\mathrm{t}=1}^{24}\mathrm{L}\mathrm{P}\mathrm{S}\left(\mathrm{t}\right)\:$$

28 $$\:\mathrm{L}\mathrm{P}\mathrm{S}\left(\mathrm{t}\right)=\left\{\begin{array}{c}1\:\:\:\:\:\:\:\:\:\:\:\:\:\:\:\:\:\:\:\:\:\:\:\:\:{\mathrm{P}}_{\mathrm{l}\mathrm{o}\mathrm{a}\mathrm{d}}\left(\mathrm{t}\right)\le\:\:{\mathrm{P}}_{\mathrm{P}\mathrm{V}}+\:{\mathrm{P}}_{\mathrm{W}\mathrm{T}}+\:{\mathrm{P}}_{\mathrm{B}\mathrm{T}}+\:{\mathrm{P}}_{\mathrm{D}\mathrm{G}}\\\:0\:\:\:\:\:\:\:\:\:\:\:\:\:\:\:\:\:\:\:\:\:\:\:\:\:{\mathrm{P}}_{\mathrm{l}\mathrm{o}\mathrm{a}\mathrm{d}}\left(\mathrm{t}\right)>\:{\mathrm{P}}_{\mathrm{P}\mathrm{V}}+\:{\mathrm{P}}_{\mathrm{W}\mathrm{T}}+\:{\mathrm{P}}_{\mathrm{B}\mathrm{T}}+\:{\mathrm{P}}_{\mathrm{D}\mathrm{G}}\end{array}\right.\:\:\:\:\:\:$$

where: the power outputs of the PV, WT, BES and DG are represented by $\:{\mathrm{P}}_{\mathrm{P}\mathrm{V}}$, $\:{\mathrm{P}}_{\mathrm{W}\mathrm{T}}$, $\:{\mathrm{P}}_{\mathrm{B}\mathrm{T}}$ and $\:{\mathrm{P}}_{\mathrm{D}\mathrm{G}}$ respectively. Each optimized parameter should remain within its allowable range of constraints as mentioned in equations from (29) to (34).

29 $$\:{\mathrm{N}}_{{\mathrm{P}\mathrm{V}}_{\mathrm{M}\mathrm{I}\mathrm{N}}}\le\:\:{\mathrm{N}}_{\mathrm{P}\mathrm{V}}\:\le\:\:{\mathrm{N}}_{{\mathrm{P}\mathrm{V}}_{\mathrm{M}\mathrm{A}\mathrm{X}}}\:\:\:\:$$

30 $$\:{\mathrm{N}}_{{\mathrm{W}\mathrm{T}}_{\mathrm{M}\mathrm{I}\mathrm{N}}}\le\:\:{\mathrm{N}}_{\mathrm{W}\mathrm{T}}\:\le\:\:{\mathrm{N}}_{{\mathrm{W}\mathrm{T}}_{\mathrm{M}\mathrm{A}\mathrm{X}}}\:\:\:$$

31 $$\:{\mathrm{N}}_{{\mathrm{B}\mathrm{T}}_{\mathrm{M}\mathrm{I}\mathrm{N}}}\le\:\:{\mathrm{N}}_{\mathrm{B}\mathrm{T}}\:\le\:\:{\mathrm{N}}_{{\mathrm{B}\mathrm{T}}_{\mathrm{M}\mathrm{A}\mathrm{X}}}\:\:\:\:$$

32 $$\:{\mathrm{N}}_{{\mathrm{D}\mathrm{G}}_{\mathrm{M}\mathrm{I}\mathrm{N}}}\le\:\:{\mathrm{N}}_{\mathrm{D}\mathrm{G}}\:\le\:\:{\mathrm{N}}_{{\mathrm{D}\mathrm{G}}_{\mathrm{M}\mathrm{A}\mathrm{X}}}\:\:\:\:\:$$

33 $$\:{\mathrm{N}}_{{\mathrm{I}\mathrm{N}\mathrm{V}}_{\mathrm{M}\mathrm{I}\mathrm{N}}}\le\:\:{\mathrm{N}}_{\mathrm{I}\mathrm{N}\mathrm{V}}\:\le\:\:{\mathrm{N}}_{{\mathrm{I}\mathrm{N}\mathrm{V}}_{\mathrm{M}\mathrm{A}\mathrm{X}}}\:\:\:\:\:\:\:$$

34 $$\:{\mathrm{N}}_{{\mathrm{C}\mathrm{C}}_{\mathrm{M}\mathrm{I}\mathrm{N}}}\le\:\:{\mathrm{N}}_{\mathrm{C}\mathrm{C}}\:\le\:\:{\mathrm{N}}_{{\mathrm{C}\mathrm{C}}_{\mathrm{M}\mathrm{A}\mathrm{X}}}\:\:\:\:\:$$

where: $\:{\mathrm{N}}_{\mathrm{P}\mathrm{V}}$, $\:{\mathrm{N}}_{\mathrm{W}\mathrm{T}}$, $\:{\mathrm{N}}_{\mathrm{B}\mathrm{T}}$, $\:{\mathrm{N}}_{\mathrm{D}\mathrm{G}}$, $\:{\mathrm{N}}_{\mathrm{I}\mathrm{N}\mathrm{V}}\:$and $\:{\mathrm{N}}_{\mathrm{C}\mathrm{C}}\:$ indicate the number of PV units, WT units, BT units, DG units, INV units and CC units respectively. The following additional constraints must also be taken into consideration as mentioned equations from (35) to (37).

35 $$\:{\mathrm{E}}_{\mathrm{L}\mathrm{O}\mathrm{A}\mathrm{D}}<{\mathrm{E}}_{\mathrm{G}\mathrm{E}\mathrm{N}}<{1.5\times\:\mathrm{E}}_{\mathrm{L}\mathrm{O}\mathrm{A}\mathrm{D}}\:$$

36 $$\:{\mathrm{P}}_{\mathrm{I}\mathrm{N}\mathrm{V}}>{\mathrm{P}}_{\mathrm{P}\mathrm{V}}+\:{\mathrm{P}}_{\mathrm{W}\mathrm{T}}$$

37 $$\:{\mathrm{P}}_{\mathrm{C}\mathrm{C}}{>\mathrm{P}}_{\mathrm{P}\mathrm{V}}\:$$

where: $\:{\mathrm{E}}_{\mathrm{L}\mathrm{O}\mathrm{A}\mathrm{D}}$ represents the required load energy and $\:{\mathrm{E}}_{\mathrm{G}\mathrm{E}\mathrm{N}}$ represents the generated power from all available sources existing in the system.

### Optimization techniques

#### Fungal growth optimizer: FGO

Fungal Growth Optimization (FGO) is a recent metaheuristic optimization algorithm developed to efficiently handle high-dimensional and nonlinear optimization problems. The algorithm updates candidate solutions using adaptive, fitness-driven mechanisms that enhance global search capability while progressively improving local convergence. These characteristics make FGO particularly suitable for techno-economic optimization of HRES, where strong coupling between system components and operational constraints exist^61^.

#### Genetic algorithm

GA is a population-based metaheuristic optimization technique inspired by the process of natural selection and the principle of survival of the fittest. In GA, a set of candidate solutions evolves iteratively through selection, crossover, and mutation operators to improve solution quality. This evolutionary process enables effective exploration of complex and nonlinear search spaces while maintaining population diversity. Due to its robustness, flexibility, and strong global search capability, GA has been widely applied to solve optimization problems in various engineering applications, including energy system optimization^62^. 

#### Grey wolf optimizer

GWO is a swarm-based metaheuristic optimization algorithm inspired by the social hierarchy and cooperative hunting behavior of grey wolves. The algorithm mimics the leadership structure of wolves (alpha, beta, delta, and omega) to guide the search process toward optimal solutions. By updating candidate solutions based on the positions of the best wolves, GWO achieves an effective balance between exploration and exploitation. Owing to its simple structure, fast convergence, and strong robustness, GWO has been widely applied in solving complex optimization problems, particularly in energy system planning and techno-economic optimization^63^. 

#### Cuckoo search optimizer

CS is a metaheuristic optimization algorithm inspired by the brood parasitism behavior of cuckoo birds. In this algorithm, each solution represents a nest, where better solutions are retained and poorer ones are replaced with a certain probability. New solutions are generated using Lévy flights, which enhance global search capability and help avoid local optima. Due to its simplicity, limited number of control parameters, and strong exploration ability, CS has been widely used for solving complex and nonlinear optimization problems, including energy system optimization^64^. 

### Case study description and weather data inputs

This research paper provides an analysis of the electrical loads in a five-star hotel having 500 rooms in the city of Safaga, Red Sea Coast, Egypt (Latitude: 26.7493° N, Longitude: 33.9836° E, Height above sea level: 423 m). The analysis takes into account hour-by-hour variation on a yearly basis, extending from July 1, 2023, until June 30, 2024. Load analysis of the hotel is based on the results of electrical loads estimations at the design stage, carried out by consulting offices that specialize in designing hotels similar in type to the one discussed herein, such as Shaker Consultancy Group, as shown in Table 7 below. Selected load classes are representative of major functions in the hotel, including guest rooms, chillers, restaurants, kitchens, services, basement services, and IT/administration services. In typical load estimations of design stage consultations, guest rooms and other equivalent facilities were usually estimated per area at a value of approximately 80 VA/m², corridors, circulation areas, services, and other equivalent functions were considered as having an equivalent value of approximately 40 VA/m², whereas basement spaces used for indoor parking were estimated per area at approximately 20 VA/m². On the other hand, calculations related to mechanical systems, low current systems, kitchen loads, and restaurant-related functional areas loads have been made using the equipment data obtained from the specialized design disciplines which designed these systems in comparable hospitality facilities in the past. Such assumptions are very common in practical design and comply with the planning methodology typically used in designing such facilities in accordance with local standards. Load variations associated with occupancy have been taken into account mostly in terms of guest room loads and hospitality loads, whereas variations associated with service area loads have been considered in accordance with the typical use of each of these zones. Seasonal variations were taken into account through changing the profile of the hotel’s loads to represent the changes in its operations and cooling load during the summer season, when these loads become higher than the rest of the year. Therefore, the profile of the hotel’s hourly loads was established based on realistic operations taking into consideration that peak loads are in summer evening times between 18:00 to 22:00, and reduced loads during overnight hours, namely, between 00:00 and 06:00.

**Table 7:** Hotel Loads.

Type	Load
Guest Rooms	2800 kVA
HVAC Chillers	2000 kVA
Restaurants	800 kVA
Kitchens	700 kVA
General Services	850 kVA
Basement Services	600 kVA
IT/Admin	550 kVA
Total connected loads	8300 kVA

#### Data Source:

**Fig. 3:**
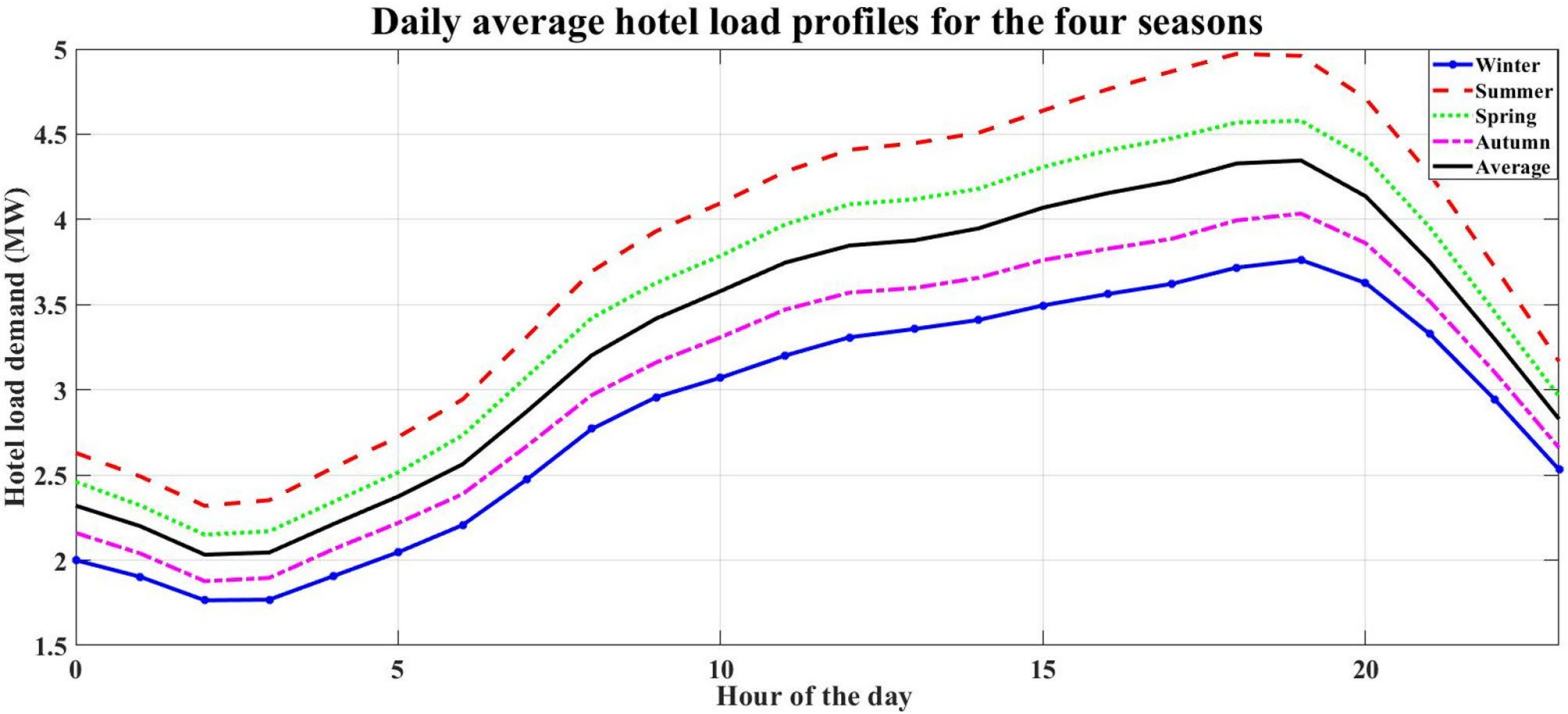
Daily average hotel load profiles for the four seasons.

**Fig. 4:**
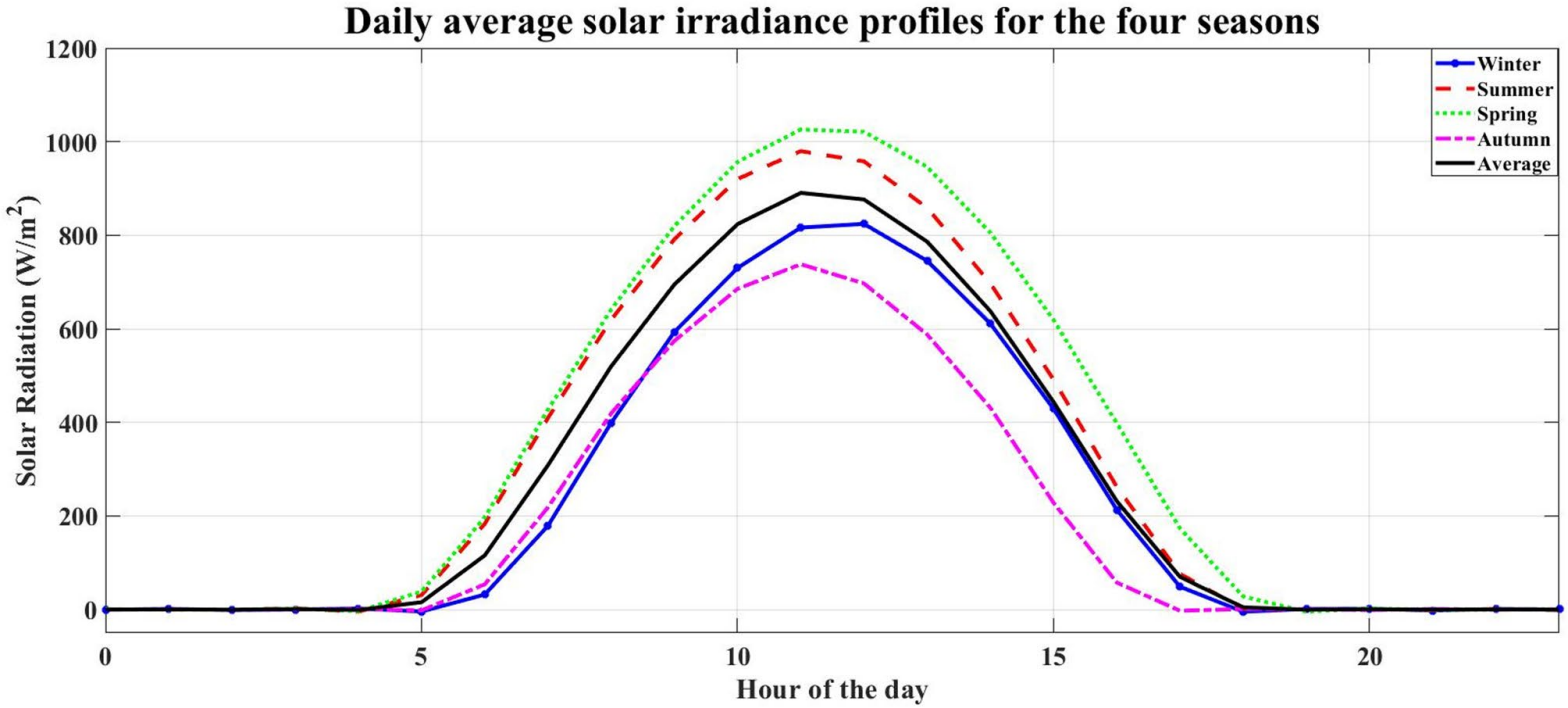
Daily average solar irradiance profiles for the four seasons.

**Fig. 5:**
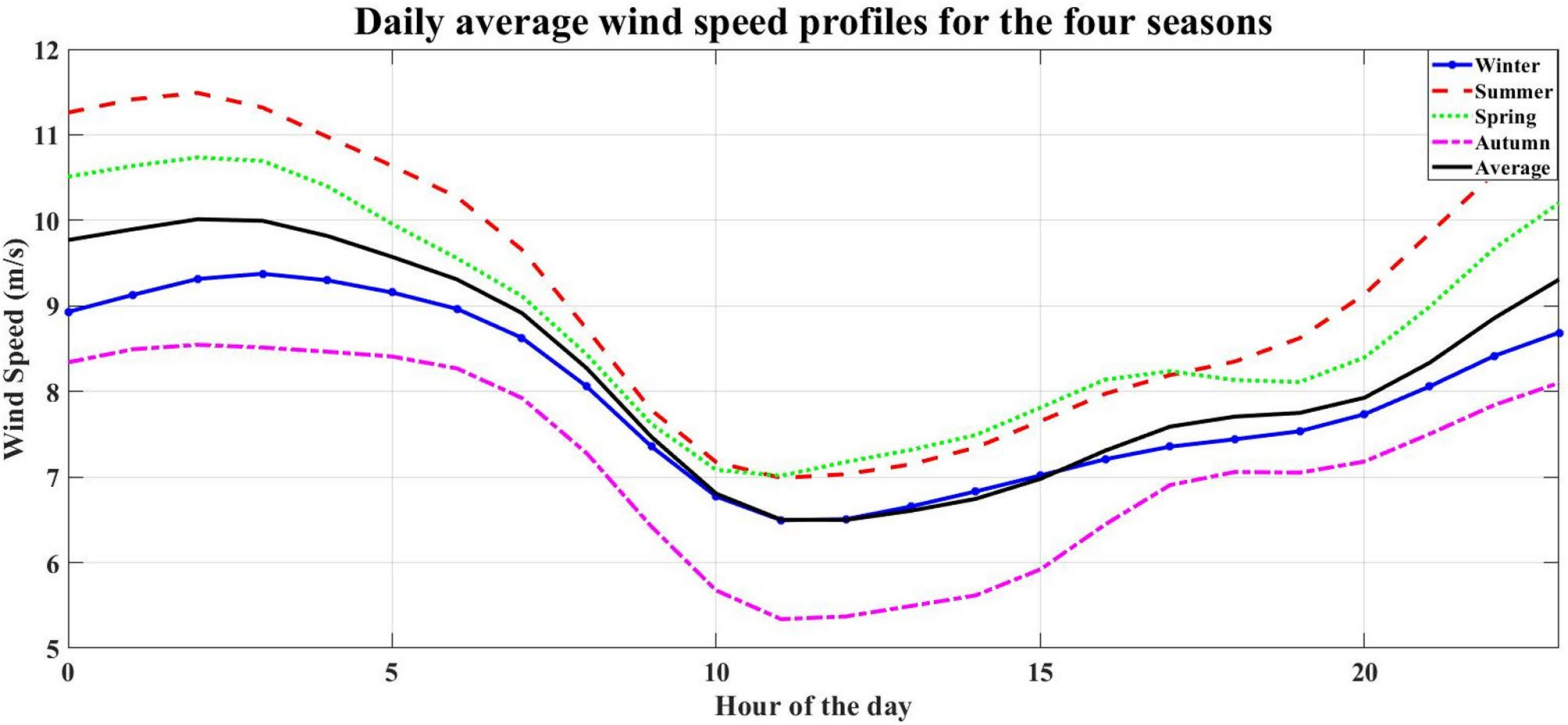
Daily average wind speed profiles for the four seasons.

**Fig. 6:**
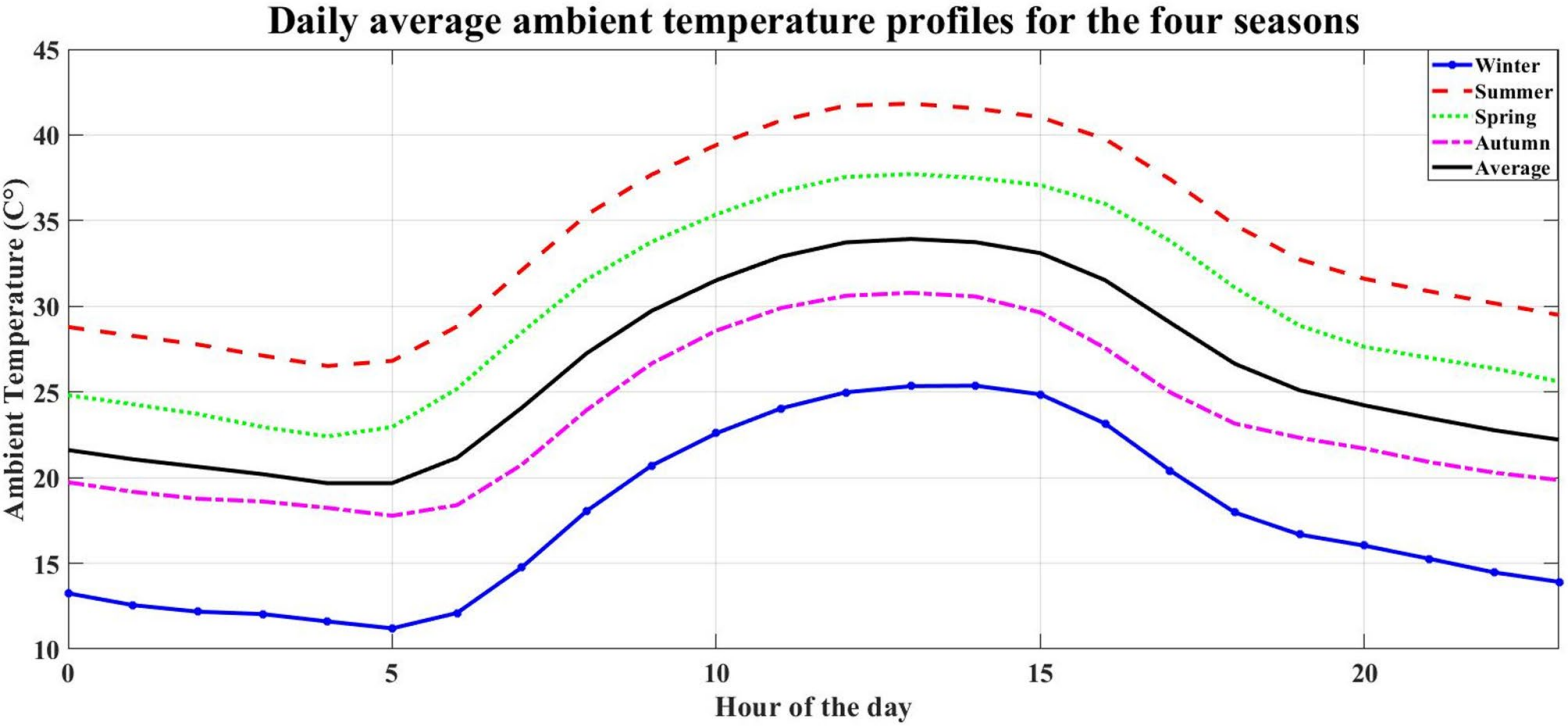
Daily average ambient temperature profiles for the four seasons.

NASA/POWER – Hourly meteorological data (07/01/2023–06/30/2024) is used to provide environmental inputs for renewable energy simulation. The data represent the solar radiation, wind speed, and temperature for each season at the study location. Figures 3, 4, 5 and 6 illustrate the daily average data for each season.

## Results and discussion

The simulation results for the proposed HRES in Safaga are thoroughly analyzed. The assessment is divided into two main frameworks: a comparative evaluation of the metaheuristic optimization algorithms and a multi-seasonal technical-economic analysis. Using GA, GWO, CS and FGO algorithms in a MATLAB 2018a environment on a personal computer, the configuration of the planned HRES was implemented. The results obtained from these metaheuristic approaches were compared. Using an hourly resolution, the energy needs were met by the simulation, using a one-year hourly dataset. For comparison purposes, the limitations of PV, WT, BT and INV were assumed to be the same for all algorithms. The system components are optimally sized based on the lowest LCOE.

### Comparative analysis of optimization algorithms

Four advanced optimization algorithms (GA, GWO, CS and FGO) were benchmarked. The comparison among the considered algorithms is presented in Table 8. The comparison among the optimization algorithms in this study is presented from a practical design perspective. Since the main objective is the seasonal sizing of an off-grid HRES for a large hotel application, greater emphasis is placed on the final economic outcome and the corresponding execution time required to obtain the solution. Therefore, the comparison is intended to identify the most suitable algorithm for the design purpose of the study rather than to provide a detailed statistical assessment of algorithmic robustness. The preference for FGO in the present study is based mainly on its competitive solution quality together with satisfactory computational performance under the tested conditions. Although GA achieved shorter execution time in the reported cases, FGO remained effective from a practical design perspective.

**Table 8:** Comparison among the algorithms.

Season	Algorithm	COE (USD/kWh)	Execution Time (s)
Autumn	FGO	0.159970	~ 23
	GA	0.159971	~ 13
	GWO	0.160135	~ 781
	CS	0.159972	~ 2418
Winter	FGO	0.124407	~ 25
	GA	0.124447	~ 13
	GWO	0.124415	~ 771
	CS	0.124413	~ 2070
Spring	FGO	0.099914	~ 24
	GA	0.099915	~ 13
	GWO	0.099922	~ 770
	CS	0.099931	~ 1724
Summer	FGO	0.098854	~ 39
	GA	0.098858	~ 21
	GWO	0.098868	~ 1339
	CS	0.098866	~ 2456

Table 8 summarizes the results, which demonstrate that all optimization algorithms consistently reached near-optimal economic solutions by achieving very close COE values throughout the four seasons. Their computational efficiency, however, showed noticeable differences. In every season, FGO maintained competitive COE values while requiring a short execution time, between roughly 23 and 39 s. While GA’s convergence behavior was less stable than FGO’s, it still produced comparable COE results with somewhat shorter runtimes. On the contrary, GWO and CS took much more time to compute—GWO over 700 s and CS sometimes over 2400 s—but with no evident improvement in COE. It shows the efficiency and effectiveness of FGO in solving seasonal sizing problems in energy-related applications, particularly when computational costs matter.

### Seasonal performance and optimal sizing

**Fig. 7:**
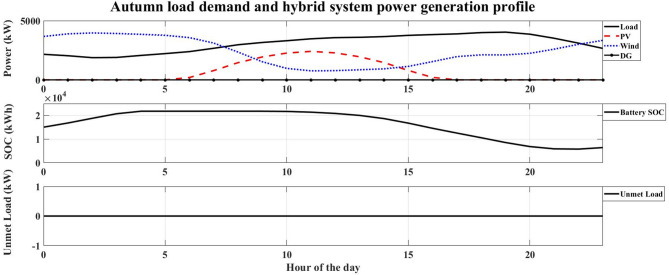
Autumn load demand and hybrid system power generation profile.

**Fig. 8:**
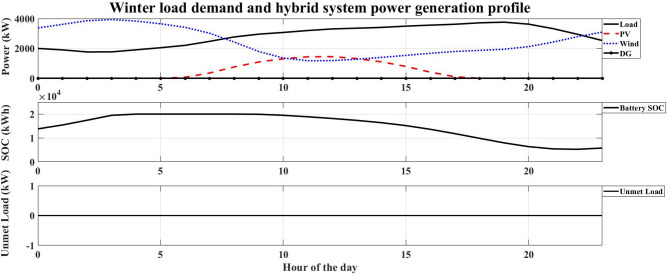
Winter load demand and hybrid system power generation profile.

**Fig. 9:**
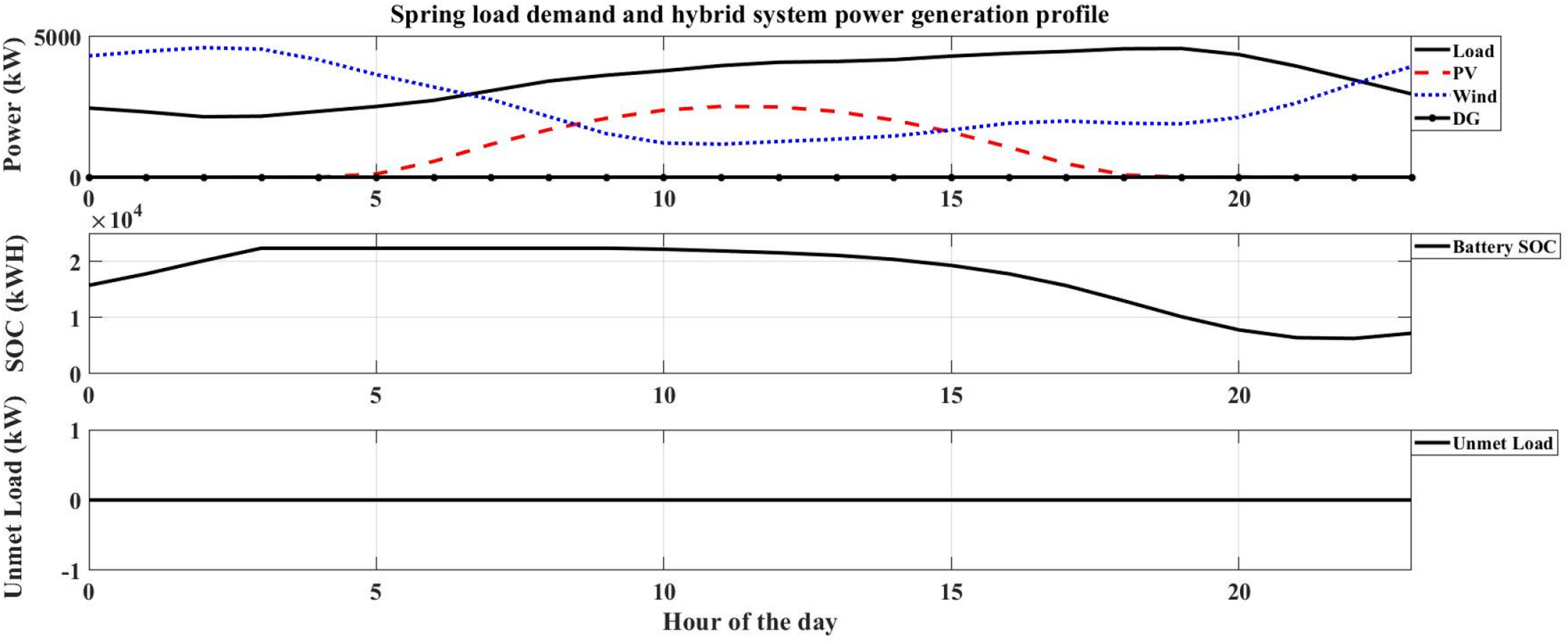
Spring load demand and hybrid system power generation profile.

**Fig. 10:**
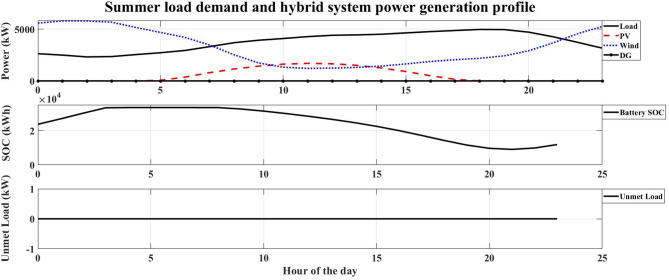
Summer load demand and hybrid system power generation profile.

The system performance was evaluated across four distinct seasons to account for the variation in the hotel’s electrical load and the intermittency of renewable resources in Safaga. The optimal number of HRES components obtained for each season is presented in Table 9, while Figs. 7, 8, 9 and 10 illustrate the relationship between the required load and the power generated by the hybrid system. The simulation was carried out at an hourly resolution, and the optimal configuration of each season was checked against its corresponding hourly load profile. The results showed that the generated and supplied power were sufficient to meet the demand throughout all simulated hours, with no unmet-load event recorded in any of the seasonal optimal cases. Therefore, the calculated LPSP remained equal to 0% for all reported solutions.

**Table 9:** Optimal number of the hybrid system components.

Season	Autumn	Winter	Spring	Summer
Number of PV Panels	24,842	13,919	19,987	14,271
Number of Wind Turbines	157	115	89	93
Number of Batteries	2721	2453	2792	4176
Number of Inverters	4	4	5	6
Number of Charge Controllers	484	306	514	343
Total Load (kWh)	6632631.49	6186085.84	7563094.93	8167764.33
Total Generation (kWh)	6635133.99	6189828.33	7563265.38	8173194.29
TNPC (M USD)	55	40	39	42
COE (USD/kWh)	0.159970	0.124407	0.099914	0.098854

- Autumn: With an LCOE of 0.15997 USD/kWh, the autumn season has the greatest LCOE. This high value shows that, in comparison to the other seasons, autumn is the least economical time to generate energy. The high LCOE is a reflection of less-than-ideal operating circumstances brought about by transitional seasonal features, where the availability of renewable energy does not perfectly match the demand profile. As a result, the unit cost of energy produced throughout this period rises.
- Winter: The LCOE in winter is 0.124407 USD/kWh, and this indicates improved utilization in the use of renewable energy sources compared to the autumn season. This means that there is a better match between energy production and load demand. Despite the limitations caused by winter conditions for some RES, the system is still at an acceptable economic level.
- Spring: The LCOE decreases further to 0.099914 USD/kWh during the spring season, indicating high economic efficiency. The low value of LCOE indicates that the system is operating under near-optimal conditions, where the generation of renewable energy is close to the demand of the system. Therefore, the spring season is one of the most economically favorable seasons to operate the system.
- Summer: Although it has the highest energy requirement, summer has the lowest LCOE of 0.098854 USD/kWh. This result indicates that the hybrid renewable energy system is most economically efficient when renewable resources are abundant. The low LCOE during peak operation conditions indicates that the HRES can provide energy at a relatively cheaper cost.

## Techno-economic and environmental discussion

From the results obtained, Safaga can be considered a very suitable site for the deployment of HRES. The use of wind and solar resources together significantly improves system performance, as these resources are complementary to each other and thus reduce the need for storage facilities.

- Resource Complementarity: The analysis shows that during the night hours and when the intensity of solar irradiance is low, wind energy emerges as the primary resource. This helps in maintaining the continuity of the system and also ensures that the state of charge of the battery is maintained, thus increasing the stability of the system.
- Sustainability Index: The achievement of an LPSP of 0% proves the technical viability of the operation of the system based on renewable energy resources, which makes the solution viable for hospitality applications. In addition to ensuring continuity of energy supply, this approach results in a significant reduction of the annual CO₂ emissions, which makes the solution compliant with the international green tourism regulations, supporting the sustainability of the hotel operations.

This change in LCOE for various seasons shows that the proposed HRES system performs best during the summer and spring seasons, as these seasons show the lowest energy costs, i.e., 0.09885 USD/kWh and 0.09991 USD/kWh, respectively. On the other hand, the least economical operating period for the system is during autumn, as this season shows the highest LCOE, i.e., 0.15997 USD/kWh.

As all the optimization methods used in this research were found to be capable of producing a feasible design, the FGO method was seen to be more efficient, as it provides a better balance between solution quality and efficiency.

A system sized for the most critical season can maintain reliable operation throughout the year, but it may also become oversized during the more favorable seasons, when renewable resources are more available and the load demand is lower. Autumn can be considered as the most critical operating period in the current case, where designing for autumn will ensure safe operation in other seasons too. But this method could lead to oversized systems for some units in spring and summer seasons, which will result in higher investment costs and low utilization of the system during that period of time. This is a common trade-off in stand-alone HRES, where reliability is usually given higher priority than minimum system size. In practical applications, alternatives such as limited grid support, demand-side management, or more diversified storage options may help reduce this effect and improve the overall economic performance. Although these alternatives were not explicitly investigated in the present study, they represent useful directions for future work.

## The reliability of the system under various failure conditions of the components

Reliability of the suggested system is tested based on probability in case of any possible failure of one or more components due to their operating state. Here, the solar cells, wind generators, batteries, and the inverter are seen as separate components that can be either in normal condition or randomly in failure during the period of simulation. The inverter is seen as a crucial single-point of failure since its failure will mean a failure of the entire system. The real amount of power that can be provided by the system is calculated depending on the working conditions of the components, in which only those components that work contribute to providing power to the load. The Monte Carlo technique is applied to randomly create failure events depending on the estimated life time of each component. Lastly, the system’s reliability can be analyzed by checking how often the system can provide continuous power to the load throughout the simulation period.

The following formulas are used to calculate the probability of failure:

38 $$\:{\uplambda\:}=\frac{1}{\mathrm{M}\mathrm{T}\mathrm{B}\mathrm{F}}\text{}\:$$

39 $$\:\mathrm{M}\mathrm{T}\mathrm{B}\mathrm{F}=8760\times\:N\:\:$$

Where: MTBF is the mean time between failures, and N is the component lifetime in years.

40 $$\:{\mathrm{P}}_{\mathrm{f}}\text{}\left(\mathrm{t}\right)=1-{\mathrm{e}}^{-{\uplambda\:}\cdot\:\mathrm{t}}\:$$

Where: t represents the mission time of the considered season.

The failure probability of each system component is calculated based on its expected lifetime using the MTBF concept. Table 10 presents the resulting failure probabilities for the main components of the proposed system, including the inverter, photovoltaic panels, wind turbines, and batteries, over the considered seasonal period.

**Table 10:** Presents the calculated failure probabilities of the main system components.

Component	$\:\mathbf{M}\mathbf{T}\mathbf{B}\mathbf{F}$		$\:{\mathbf{P}}_{\mathbf{f}}\mathbf{}\left(\mathbf{t}\right)$
Inverter	15 Years	131,400 h	0.016304
Solar Panels	25 Years	219,000 h	0.009815
Wind turbines	25 Years	219,000 h	0.009815
Batteries	10 Years	87,600 h	0.024356

The system reliability is evaluated using the following relations:

41 $$\:{\mathrm{S}}_{\mathrm{x}}\left(\mathrm{t}\right)=\left\{\begin{array}{c}1\:\:\:\:\:\:\:\:\:\:\:\:\:\:\:component\:is\:operating\\\:0\:\:\:\:\:\:\:\:\:\:\:\:\:\:\:\:\:\:\:\:\:\:\:\:\:\:\:component\:failed\end{array}\right.\:\:$$

Where: $\:{\mathrm{S}}_{\mathrm{I}\mathrm{N}\mathrm{V}}$, $\:{\mathrm{S}}_{\mathrm{P}\mathrm{V}}$, $\:{\mathrm{S}}_{\mathrm{W}\mathrm{T}}$, and $\:{\mathrm{S}}_{\mathrm{B}\mathrm{T}}$ represent the operating states of the inverter, PV panels, wind turbines, and batteries, respectively, and $\:i$ refers to the number of Monte Carlo simulations.

42 $$\:{\mathrm{S}}_{\mathrm{I}\mathrm{N}\mathrm{V}}\text{}\left(\mathrm{t}\right)=0\:,\:{\mathrm{P}}_{\mathrm{T}\mathrm{O}\mathrm{T}\mathrm{A}\mathrm{L}}\left(\mathrm{t}\right)=0\:\:$$

Since the inverter is considered a single point of failure, its failure leads to zero total output power.

43 $$\:{\mathrm{P}}_{{\mathrm{W}\mathrm{T}}_{\mathrm{A}\mathrm{C}\mathrm{T}}}\left(\mathrm{t}\right)={\mathrm{S}}_{\mathrm{W}\mathrm{T}}\text{}\left(\mathrm{t}\right)\cdot\:{\mathrm{P}}_{\mathrm{W}\mathrm{T}}\left(\mathrm{t}\right)$$

44 $$\:{\mathrm{P}}_{{\mathrm{P}\mathrm{V}}_{\mathrm{A}\mathrm{C}\mathrm{T}}}\left(\mathrm{t}\right)={\mathrm{S}}_{\mathrm{P}\mathrm{V}}\text{}\left(\mathrm{t}\right)\cdot\:{\mathrm{P}}_{\mathrm{P}\mathrm{V}}\left(\mathrm{t}\right)\:\:$$

45 $$\:{\mathrm{P}}_{{\mathrm{B}\mathrm{T}}_{\mathrm{A}\mathrm{C}\mathrm{T}}}\left(\mathrm{t}\right)={\mathrm{S}}_{\mathrm{B}\mathrm{T}}\text{}\left(\mathrm{t}\right)\cdot\:{\mathrm{P}}_{\mathrm{B}\mathrm{T}}\left(\mathrm{t}\right)\:$$

46 $$\:{\mathrm{P}}_{\mathrm{T}\mathrm{O}\mathrm{T}\mathrm{A}\mathrm{L}}\left(\mathrm{t}\right)=\:{\mathrm{P}}_{\mathrm{P}{\mathrm{V}}_{\mathrm{A}\mathrm{C}\mathrm{T}}}\left(\mathrm{t}\right)+\:{\mathrm{P}}_{\mathrm{W}{\mathrm{T}}_{\mathrm{A}\mathrm{C}\mathrm{T}}}\left(\mathrm{t}\right)+\:{\mathrm{P}}_{{\mathrm{B}\mathrm{T}}_{\mathrm{A}\mathrm{C}\mathrm{T}}}\left(\mathrm{t}\right)$$

47 $$\:\mathrm{L}\mathrm{P}\mathrm{S}\left(\mathrm{t}\right)=\left\{\begin{array}{c}1\:\:\:\:\:\:\:\:\:\:\:\:\:\:\:\:\:\:\:\:{\mathrm{P}}_{\mathrm{l}\mathrm{o}\mathrm{a}\mathrm{d}}\left(\mathrm{t}\right)>\:{\mathrm{P}}_{\mathrm{T}\mathrm{O}\mathrm{T}\mathrm{A}\mathrm{L}}\\\:0\:\:\:\:\:\:\:\:\:\:\:\:\:\:\:\:\:\:\:\:\:\:\:\:\:\:\:\:\:\:\:\:\:\:Otherwise\end{array}\right.\:$$

48 $$\:\mathrm{L}\mathrm{P}\mathrm{S}\mathrm{P}=\frac{1}{\mathrm{T}}{\sum\:}_{\mathrm{t}=1}^{\mathrm{T}}\mathrm{L}\mathrm{P}\mathrm{S}\left(\mathrm{t}\right)$$

49 $$\:\mathrm{R}\mathrm{e}\mathrm{l}\mathrm{i}\mathrm{a}\mathrm{b}\mathrm{i}\mathrm{l}\mathrm{i}\mathrm{t}\mathrm{y}=1-\mathrm{L}\mathrm{P}\mathrm{S}\mathrm{P}\:\:\:$$

Based on this procedure, the mean values of LPSP and system reliability are calculated for each season. Table 11. summarizes the obtained mean LPSP and reliability values for each season, providing a clear comparison of the system performance under component failure conditions.

**Table 11:** The obtained mean LPSP and reliability values for each season.

Season	Autumn	Winter	Spring	Summer
Mean LPSP	0.022991	0.023125	0.011956	0.016339
Mean Reliability	0.977009	0.976875	0.988044	0.983661

The Monte Carlo reliability analysis indicates that the proposed system maintains high reliability in all studied seasons under possible component failure conditions. The best result is obtained in spring, where the Mean LPSP is 0.011956 and the Mean Reliability reaches 0.988044, indicating that nearly 98.8% of the load can still be satisfied. Moreover, summer demonstrates high reliability with Mean Reliability being 0.983661, while the values for autumn and winter are slightly less, yet almost the same at 0.977009 and 0.976875, correspondingly. Thus, it may be concluded that the developed HRES is robust enough to work under uncertainty conditions and seasonal variations of reliability.

## Sensitivity analysis

In order to comprehend the economic reaction of the suggested HRES to the variations in the mentioned inputs, a sensitivity analysis was carried out regarding the cost of PV panels, wind turbines, inflation rate, and interest rate. Sensitivity analysis can be considered an effective technique that helps evaluate the effects of these parameters on the COE and the sizing solutions. The detailed findings of this analysis are presented in Tables 12, 13, 14 and 15, whereas Fig. 1 shows the sensitivity graph for the investigated inputs.

**Table 12:** Seasonal sensitivity analysis of PV panel cost variation on system size and COE.

Percentage of PV panels cost	Season	Autumn	Winter	Spring	Summer
50%	Number of PV Panels	45,196	26,332	28,264	28,770
	Number of Wind Turbines	122	96	77	77
	COE (USD/kWh)	0.137517	0.108607	0.084957	0.083229
75%	Number of PV Panels	28,292	19,805	22,742	26,041
	Number of Wind Turbines	151	106	85	80
	COE (USD/kWh)	0.150136	0.117341	0.092733	0.091519
100%	Number of PV Panels	24,842	13,919	19,987	14,271
	Number of Wind Turbines	157	115	89	93
	COE (USD/kWh)	0.159970	0.124407	0.099914	0.098854
125%	Number of PV Panels	1563	100	2154	3
	Number of Wind Turbines	197	136	115	109
	COE (USD/kWh)	0.161578	0.125445	0.104289	0.099545
150%	Number of PV Panels	0	92	16	2
	Number of Wind Turbines	200	136	118	109
	COE (USD/kWh)	0.161694	0.125480	0.104519	0.099544

**Table 13:** Seasonal sensitivity analysis of WT cost variation on system size and COE.

Percentage of Wind Turbines cost	Season	Autumn	Winter	Spring	Summer
50%	Number of PV Panels	0	0	0	0
	Number of Wind Turbines	200	172	173	160
	COE (USD/kWh)	0.102737	0.082008	0.068479	0.069015
75%	Number of PV Panels	1552	121	11,110	0
	Number of Wind Turbines	197	136	103	124
	COE (USD/kWh)	0.131965	0.103931	0.088090	0.086255
100%	Number of PV Panels	24,842	13,919	19,987	14,271
	Number of Wind Turbines	157	115	89	93
	COE (USD/kWh)	0.159970	0.124407	0.099914	0.098854
125%	Number of PV Panels	27,126	18,510	22,074	26,234
	Number of Wind Turbines	153	108	86	80
	COE (USD/kWh)	0.182930	0.141919	0.111068	0.109056
150%	Number of PV Panels	30,619	20,433	22,742	26,061
	Number of Wind Turbines	147	105	85	80
	COE (USD/kWh)	0.204972	0.158647	0.122084	0.118516

**Table 14:** Seasonal sensitivity analysis of inflation rate variation on system size and COE.

Percentage of inflation rate	Season	Autumn	Winter	Spring	Summer
50%	Number of PV Panels	24,276	13,869	19,372	14,558
	Number of Wind Turbines	158	115	90	93
	COE (USD/kWh)	0.167766	0.127418	0.104853	0.103917
75%	Number of PV Panels	24,238	13,942	19,415	14,462
	Number of Wind Turbines	158	115	90	93
	COE (USD/kWh)	0.163706	0.125940	0.102329	0.101360
100%	Number of PV Panels	24,842	13,919	19,987	14,271
	Number of Wind Turbines	157	115	89	93
	COE (USD/kWh)	0.159970	0.124407	0.099914	0.098854
125%	Number of PV Panels	24,247	13,932	19,308	14,554
	Number of Wind Turbines	158	115	90	93
	COE (USD/kWh)	0.156394	0.121678	0.097706	0.096555
150%	Number of PV Panels	24,253	13,901	19,408	14,214
	Number of Wind Turbines	158	115	90	93
	COE (USD/kWh)	0.153102	0.119005	0.095548	0.094304

**Table 15:** Seasonal sensitivity analysis of interest rate variation on system size and COE.

Percentage of interest rate	Season	Autumn	Winter	Spring	Summer
50%	Number of PV Panels	6183	3384	19,435	566
	Number of Wind Turbines	189	131	90	108
	COE (USD/kWh)	0.137875	0.106868	0.085664	0.083263
75%	Number of PV Panels	18,425	12,647	19,388	14,304
	Number of Wind Turbines	168	117	90	93
	COE (USD/kWh)	0.147484	0.114624	0.091936	0.090538
100%	Number of PV Panels	24,842	13,919	19,987	14,271
	Number of Wind Turbines	157	115	89	93
	COE (USD/kWh)	0.159970	0.124407	0.099914	0.098854
125%	Number of PV Panels	24,982	14,623	20,158	14,347
	Number of Wind Turbines	157	114	89	93
	COE (USD/kWh)	0.175080	0.136315	0.109452	0.108540
150%	Number of PV Panels	24,962	14,573	20,206	14,670
	Number of Wind Turbines	157	116	89	93
	COE (USD/kWh)	0.192331	0.149806	0.120247	0.119392

**Fig. 11:**
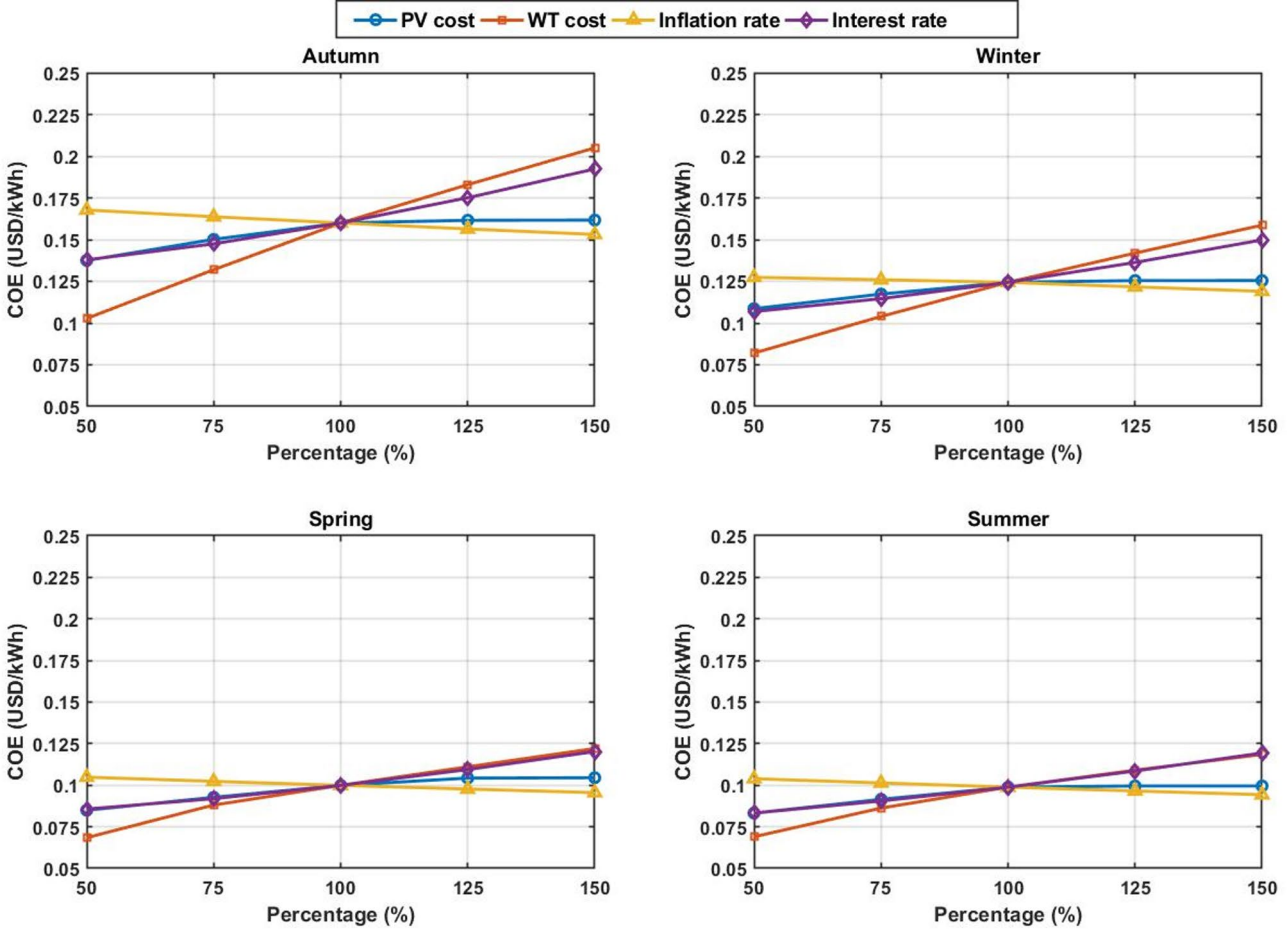
The Variation of COE with percentage changes in PV panel cost, WT cost, inflation rate, and interest rate for autumn, winter, spring, and summer.

The output values obtained from the simulation and provided in Tables 12, 13, 14 and 15 highlight the change in the value of system sizing and COE based on changes in the percent of the parameters analyzed. Tables 12 and 13 reflect the effects of the PV panel and wind turbine costs on the number of PV panels, wind turbines, and COE during each season. Likewise, Tables 14 and 15 reflect the effects of changes in inflation and interest rates on the same factors (see Fig. 11).

According to the sensitivity analysis, the COE of the suggested system is influenced differently by the economic factors analyzed. From all these factors, the wind turbine price has the greatest impact on the COE for all seasons, while the interest rate is second. The PV panel price, however, does not affect the COE greatly but affects the system optimum size. In contrast, inflation rate has the least overall influence on both the COE and the selected configuration. For example, increasing wind turbine cost from 50% to 150% raises the autumn COE from 0.102737 to 0.204972 USD/kWh, which represents the largest variation among all considered parameters. On the other hand, increasing PV panel cost over the same range changes the autumn COE from 0.137517 to 0.161694 USD/kWh, while causing a much larger shift in the optimal numbers of PV panels and wind turbines. The interest rate also has a significant effect because of the capital-intensive nature of the proposed system, whereas inflation rate causes only limited changes in both the economic results and the optimal sizing. In all cases, autumn remains the most critical season and summer remains the most favorable one, which confirms the consistency of the main seasonal optimization results.

## Conclusions

In this study, a comprehensive techno-economic optimization framework was developed for the design of an off-grid HRES supplying a large coastal hotel in Safaga, Egypt. The proposed system integrates photovoltaic panels, wind turbines, and battery storage, with a diesel generator used only as a backup source under critical conditions.

- A comparative evaluation of four metaheuristic optimization algorithms was conducted under identical modeling conditions. The findings indicated that all of the algorithms were able to generate almost optimal solutions with very similar COEs, while Fungal Growth Optimizer (FGO) exhibited a good trade-off between accuracy and speed, thus being applicable in engineering design.
- From the seasonal analysis, it was noted that variations in load demand and presence of renewable energy resources greatly affect the performance of the system. From the findings obtained, it is noted that the economic performance of the system is highest during summer and spring seasons, having COE of 0.098854 USD/kWh and 0.099914 USD/kWh, respectively, and the worst performing season is autumn with COE of 0.159970 USD/kWh.
- Furthermore, the reliability analysis carried out using Monte Carlo Simulation shows that the developed model exhibits high reliability even in situations of component failures, having mean reliability values between 97.69% and 98.80%. This indicates that the model has high reliability even when there is uncertainty regarding component availability.
- Moreover, the sensitivity analysis indicated that the performance of the system was affected by the changes in both economic and technical factors. The findings indicated that the cost of wind turbines had the highest impact on COE, whereas the cost of PV was more influential on the system design. However, the economic factors such as inflation rate and interest rates had a greater impact on the cost of energy production.

In conclusion, the results of the present study validate the applicability of the designed HRES as an efficient and economical power generation technology, contributing to sustainable development by minimizing the consumption of non-renewable energy sources. Further research could concentrate on the incorporation of innovative storage systems and the assessment of grid-connected systems to optimize performance and financial viability.

## Data Availability

The datasets analyzed during the current study are available from the corresponding author on reasonable request.
